# Social Curiosity and Gossip: Related but Different Drives of Social Functioning

**DOI:** 10.1371/journal.pone.0069996

**Published:** 2013-07-31

**Authors:** Freda-Marie Hartung, Britta Renner

**Affiliations:** Department of Psychology, Psychological Assessment and Health Psychology, University of Konstanz, Konstanz, Germany; George Mason University/Krasnow Institute for Advanced Study, United States of America

## Abstract

The present online-questionnaire study examined two fundamental social behaviors, social curiosity and gossip, and their interrelations in an English (*n* = 218) and a German sample (*n* = 152). Analyses showed that both samples believed that they are less gossipy but more curious than their peers. Multidimensional SEM of self and trait conceptions indicated that social curiosity and gossip are related constructs but with different patterns of social functions. Gossip appears to serve predominantly entertainment purposes whereas social curiosity appears to be more driven by a general interest in gathering information about how other people feel, think, and behave and the need to belong. Relationships to other personality traits (N, E, O) provided additional evidence for divergent validity. The needs for gathering and disseminating social information might represent two interlinked but different drives of cultural learning.

## Introduction

Humans live in a complex social world, and building and using networks of relationships represent a central task [1]. In order to function efficiently in a changing and complex social environment, humans require information about those around them [2]. Therefore, both social curiosity and the tendency to gossip are at the heart of social and cultural life [3]–[5].

Gossip has been broadly defined as conversation about social and personal topics and it has been argued that it is the central player in the evolutionary story of human intelligence and social life [2]–[4]. Similarly, curiosity has been defined as the basic drive to learn, which sets the stage for development and learning ([6]–[8], but see [9]). Thus, both social curiosity and the tendency to gossip might facilitate and direct learning and understanding of social information. Besides the apparent overlap between the two concepts, however, they may also tap into different aspects of social life and may have different social functions.

The present study is the first to assess individual differences in both social curiosity and the tendency to gossip as well as determine the structural relationship between the two constructs and their relation to other trait concepts of curiosity and personality measures. In order to corroborate the structural assumptions, the study was conducted with two samples from English- and German-speaking countries.

### Conceptions of Curiosity

Curiosity has been conceptualized as desire for new information and knowledge [10]–[14]. Social curiosity, a facet of curiosity, has been conceptualized as the general interest in gaining new social information motivating exploratory behaviors [15], [16]. Accordingly, social curiosity is a motive-behavior system entailing two different aspects: a general interest in the acquisition of new information about how other people behave, act and feel (motive) and an interest in interpersonal information that is obtained through exploratory behaviors (behavior). Turning to lay conceptions shows that social curiosity is appraised as a rather desirable trait. Older as well as younger adults rated themselves on average as being more curious than an average peer. These self-ratings of curiosity were positively related to measures of trait social curiosity, indicating that lay and scientific conceptions overlap substantially [15].

### Conceptions of Gossip

For the most part, authors agree that gossip refers to talk in an evaluative way (positive or negative) about absent third parties [2]. Other suggested definitions of the phenomenon revolve around this summary, for example, including conversations when the gossipee is present [4] or that the talk must be negative or malicious [17]. In general, gossip appears to be a widely spread phenomenon and almost inevitable when two or more people meet. Observational studies assessing the content of conversations in public setting showed that about two thirds of adult conversations involved gossip [18], [19]. Levin and Arlucke [19], for example, observed that about 68% of all conversations at a university cafeteria were about absent persons. In a similar vein, Dunbar, Marriott, and Duncan [18] found that for two thirds of the time, and for both genders, conversations were centered on social topics. Conversely, when asking people to report directly about how much they gossiped in a conversation, men as well as women reported that they do so less than 30% of the time ([20] as cited in [21]). The discrepancy between observational and self-report studies on gossip may stem from underreporting due to respondents’ awareness that gossip is an undesirable mode of behavior [21]. Supporting this notion, Litman and Pezzo [22] found a negative correlation between gossip and social desirability. However, another possible explanation might be that the everyday understanding of the term gossip is different from the construct as used by researchers [2].

### Social Curiosity and Gossip: Related but Different Social Functions

Curiosity has been recognized as a driving force in human development and learning developed in the course of evolution (cf. [14], [23]). This interest in novelty appears to be a core determinant of individual differences in intelligence and development over the life span [6], [8]. Thus, a core function of curiosity is acquiring information to foster learning and development. Another function of social curiosity might be to form interpersonal attachments and facilitate feelings of belonging. Consistent with this notion, social curiosity is positively related to social functioning, such as extraversion and social competence. In particular, people scoring high on interpersonal curiosity are more likely to be socially competent, sociable, and able to build networks of relationships that provide support in the face of stressful life events ([15], see also [24]). A third function of social curiosity may be a reflection of the need to live in a predictable and controllable social world. For instance, Swann, Stephenson, and Pittman [25] showed that individuals who had recently been deprived of control demanded more diagnostic information about a person they were due to interview than individuals who had not been deprived of control. Thus, social curiosity might serve three different motives related to social functioning: acquiring information, building and establishing relationships, and control of the social environment.

Similar functions have been postulated for gossip. Foster [2] summarized four social functions of gossip: Information, friendship/intimacy, influence, and entertainment. Specifically, the “information” function refers to gossiping as a mechanism of information exchange fostering cultural learning [3]. “Friendship/intimacy” represents gossiping as a bonding mechanism in dyadic interchanges [4], [26]. Sharing gossip is a way to socialize and to build relationships through the sharing of norms, the disclosure of trusted information, and the exclusion of outsiders. Moreover, it has been proposed that gossip serves as an effective policing device for controlling free riders and social cheats (e.g., [4], [17], [27]–[29]). That gossip has a high entertainment and recreational value becomes immediately apparent when observing people engaged in a casual conversation. People often explain their involvement in gossip with the immediacy of entertainment and pleasure [22], [30].

Thus, social curiosity and gossip appear to highly overlap in terms of social functions. However, from a theoretical perspective two differences emerge. Firstly, gossip is a behavior, whereas social curiosity describes a motive-behavior system. The “drive to know” as Kagan [31] characterized curiosity, motivates exploratory behavior in order to satisfy this desire. However, various exploratory strategies might serve to satisfy social curiosity. For instance, people may take active steps to acquire information about other persons, e.g., asking them probing questions in the hope of unearthing hidden secrets. At other times, people might also use covert, or even privacy-violating strategies, such as eavesdropping or observing people surreptitiously. Socially anxious people, for example, tend more often to use visual inspection or eavesdropping as an exploratory strategy [8], [15]. Gossiping represents a third type of exploratory strategy for gaining social information which is less intrusive than directly asking the target person and more open than surreptitious observation or eavesdropping. Accordingly, social curiosity represents a motive or desire and gossip a strategy to satisfy the desire of social curiosity.

Secondly, entertainment appears to be more a social function of gossip than of social curiosity. Many people pass time gossiping simply for the sake of amusement. Conversely, entertainment might be a by-product of exploratory behaviors and learning in the realm of social curiosity but presumably not a core function.

### The Present Study

The aim of the present study was to examine the structural relationships between social curiosity and gossip from two perspectives. First, previous studies suggest that lay and scientific conceptions of curiosity and gossip differ. Therefore, the present study determined (a) the relationship between lay conceptions of curiosity and gossip and (b) their relation to scientific trait conceptions of curiosity and gossip in order to identify differences and similarities between lay and scientific conceptions of gossip and curiosity.

Second, current theoretical conceptions allow two interpretations of gossip: Gossip might be conceptualized as an exploratory behavior which might serve to satisfy social curiosity, thus, representing one facet of social curiosity. Conversely, gossip may represent a distinctive concept that overlaps social curiosity in terms of social functions such as learning, relationship building, and social control but diverge in terms of entertainment. In order to determine whether (a) gossip represents one facet of social curiosity or (b) gossip and social curiosity represent two distinguishable concepts that serve similar social functions, the structural relationship between both constructs was determined using structural equation modelling (SEM). Additionally, their relation to other trait curiosity and personality measures was determined in order to examine the convergent and divergent validity of the found structural pattern.

## Methods

### Ethic Statement

Participants were invited to fill out an online questionnaire designed to find out more about the way people perceive themselves. The questionnaire was undertaken with the informed consent of each subject. We strictly followed the guidelines of the German Psychological Society (Deutsche Gesellschaft für Psychologie, DGPs) for conducting psychological studies (http://www.dgps.de/dgps/aufgaben/003.php; see paragraph C.III). These guidelines are similar to those of the American Psychological Association (APA). Hence, all participants read detailed instructions at the beginning of the questionnaire according to the ethics guidelines of the German Psychological Society (ethics board of the DGPs: http://www.dgps.de/dgps/aufgaben/ethikrl2004.pdf). Participants only began the questionnaire after consenting to these conditions. The instruction page serves as documentation of their informed consent. The study conformed to the Declaration of Helsinki and the ethics guidelines of the German Psychological Society and, thus, did not require any additional ethics approval (see also [32]). All data were analyzed anonymously.

### Participants

In total, 370 participants between the ages of 16 and 77 (69% women) were recruited. Two hundred eighteen participants came from English speaking countries (USA, Canada, Australia, United Kingdom, Ireland, New Zealand); 78% women; mean age 25 years (*SD* = 9.6). Hundred fifty two participants came from German speaking countries (Germany, Austria, Switzerland); 57% female; mean age 30 years (*SD* = 9.7). Ten participants had 5% or less missing values. According to standard procedures, missing items were imputed prior to forming scales by averaging the items that remained (cf. [33]).

### Instruments and Procedure

Each participant filled in an online-questionnaire including seven scales (cf., Table 1). Participants were instructed to rate how they “generally perceive themselves” on a 4-point Likert scale ranging from 1 (*strongly disagree*) to 4 (s*trongly agree*). Approximately 25 to 35 minutes were required to fill in the questionnaire.

**Table 1:** Means, Standard Deviations, Cronbach’s Alpha Coefficients, and Effect Sizes for Sample Differences (Pearson’s r) for the English Sample (*n* = 218) and German Sample (*n* = 152).

		Total Sample		English Sample		German Sample		
		(*N = *370)		(*n = *218)		(*n = *152)		
		Mean (*SD*)	*α*	Mean (*SD*)	*α*	Mean (*SD*)	*α*	*d*
Social	SCS	27.91 (4.22)	.77	27.95 (4.46)	.80	27.84 (3.88)	.72	.03
Curiosity	SCS-G	15.36 (2.36)	.75	15.26 (2.44)	.77	15.51 (2.23)	.72	.11
	SCS-C	12.54 (2.77)	.70	12.69 (2.84)	.72	12.32 (2.67)	.68	.13
Trait	EC	30.09 (4.80)	.87	29.42 (5.14)	.89	31.06 (4.08)	.82	.35***
Curiosity	CEI	19.71 (2.97)	.73	19.61 (3.18)	.75	19.85 (2.64)	.68	.08
Gossip	GFQ	60.62 (8.28)	.85	60.56 (8.76)	.86	60.71 (7.56)	.83	.02
	GFQ-I	16.84 (2.57)	.62	16.80 (2.62)	.63	16.91 (2.51)	.62	.04
	GFQ-F	15.30 (2.65)	.67	15.37 (2.76)	.69	15.19 (2.51)	.66	.07
	GFQ-If	14.16 (2.42)	.63	14.21 (2.55)	.63	14.09 (2.23)	.63	.05
	GFQ-E	14.32 (2.84)	.68	14.18 (2.88)	.68	14.52 (2.77)	.70	.12
NEO	E	32.33 (5.42)	.83	32.52 (5.86)	.84	32.06 (4.70)	.79	.08
	O	35.31 (4.66)	.75	34.50 (5.01)	.77	36.47 (3.83)	.65	.43***
	N	29.85 (6.22)	.87	29.82 (6.29)	.86	27.28 (5.70)	.86	.42***

*Notes*: SCS = Social Curiosity Scale; SCS-G = Subscale Social Curiosity-General; SCS-C = Subscale Social Curiosity-Covert; EC = Epistemic Curiosity Scale; CEI = Curiosity and Exploration Inventory – Trait Form; GFQ = Gossip Function Questionnaire; GFQ-I = Gossip Function Questionnaire-Information Subscale; GFQ-F = Gossip Function Questionnaire-Friendship Subscale; GFQ-If = Gossip Function Questionnaire-Influence Subscale; GFQ-E = Gossip Function Questionnaire-Entertainment Subscale; N = Neuroticism; E = Extraversion; O = Openness. *** *ts* >3; *p*<.001.

#### Social curiosity scale

The Social Curiosity Scale (SCS [15]) contains 10 items assessing a broad interest in the acquisition of new information about how other people behave, think, and feel which motivate exploratory behaviors. The subscale *“General Social Curiosity*” describes curiosity in other people’s habits, feelings, and thinking (e.g., “When I meet a new person, I am interested in learning more about him/her.”). The subscale, *“Covert Social Curiosity”*, includes items such as eavesdropping on conversations or observing people surreptitiously (e.g., “When on the train, I like listening to other people’s conversations.”). For the present study, the German Social Curiosity Scale was translated into English by two bilingual and bicultural individuals, and the authors using the parallel blind technique [34]. The English version of the SCS exhibited satisfactory reliability in this study with α* = *.80 (English sample) and α* = *.72 (German sample), which is comparable to previous research using the German version of the SCS scale with α = .81 [15], [16].

#### Epistemic curiosity inventory

The Epistemic Curiosity Inventory (EC [13]) consists of 10 items which measure interest in exploring new ideas and figuring out how things work (e.g., “When I see a complicated piece of machinery, I like to ask someone how it works.”). The EC scale exhibited good reliability in this study with α* = *.89 (English sample) and α* = *.82 (German sample), which is comparable to previous research using the EC scale that ranged between α = .81 and α = .85 [12], [13].

#### Curiosity and exploration inventory

The 7-item trait version of the Curiosity and Exploration Inventory (CEI; [35], [36]) assesses two dimensions of trait curiosity: (a) exploration, which refers to appetitive strivings for novelty and challenge (e.g., “I would describe myself as someone who actively seeks as much information as I can in a new situation.”), and (b) absorption, which refers to flow-like activity engagement (“When I am actively interested in something, it takes a great deal to interrupt me.”). The CEI scale alpha coefficients were at an acceptable level for both the English (α = .75) and German samples (α = .68), and comparable to the previous studies, with alpha coefficients ranging from.72 to.80 [35], [36].

#### Gossip

The 24-item Gossip Functions Questionnaire (GFQ [2]) consists of four six-item subscales assessing social functions of conversations: (a) “GFQ-Information”, which describes social conversations as means of gathering or disseminating information (e.g., “Generally, I try to figure out what is going on in the lives of people around me.”), (b) “GFQ-Friendship” subscale refers both to dyadic interchanges and to the way in which social conversations bring people together via sharing information (e.g., “Talking about the personal lives of other people makes me feel in touch with my social circle.”), (c) “GFQ-Influence” describes social exchange as an informal social mechanism for controlling free riders and social cheats (e.g., “When someone does something inappropriate, I think others should know so the person will be less likely to do it again.”), and (d) “GFQ-Entertainment” refers to the pleasure and amusement people derive from conversations (e.g., “I don’t have to know whether talk about people is true or not to enjoy the activity.”). The GFQ scale had good reliability in this study with α = .86 (English sample) and α* = *.83 (German sample). The four subscales, however, yielded lower alpha coefficients with GFQ-Information α = .63 and α = .62; GFQ-Friendship α = .69 and α = .66; GFQ-Influence α = .63; and GFQ-Entertainment α = .68 and α = .70 for the English and German sample, respectively. In previous studies, internal consistency varied between.81 and.64 [2], [37].

#### Personality traits

Neuroticism, Extraversion, and Openness were assessed using the 12-item scales from the NEO-FFI [38]. Coefficient alphas for the three traits obtained in the current study were as follows: Neuroticism (α = .86 for the English and German sample), Extraversion (α = .84 for the English sample and.79 for the German sample), and Openness (α = .77 for the English sample and.65 for the German sample). All alphas were comparable to those reported by McCrae and Costa ([39]; Neuroticism α = .86; Extraversion α = .80; and Openness α = .75). Data sets of 63 participants on the Neuroticism scale are missing due to technical problems during the assessment.

#### Lay conceptions of curiosity and gossip

In order to capture lay conceptions of curiosity and gossip participants were asked to rate themselves for the personality traits curiosity and gossiping (e.g., “I see myself as someone who is curious.”). Answers for absolute ratings were given on a 4-point scale ranging from “strongly disagree” (1) to “strongly agree” (4). In addition, participants were asked to judge themselves on the two personality traits compared to an average peer of the same sex. Answers for comparative ratings were given on a 7-point rating scale ranging from “much below average” (1), “average” (4) to “much above average” (7). Comparative ratings were recoded into “much below average” (−3), “average” (0) to “much above average” (+3).

## Results

### Lay Conceptions of Social Curiosity and Gossip

**Figure 1:**
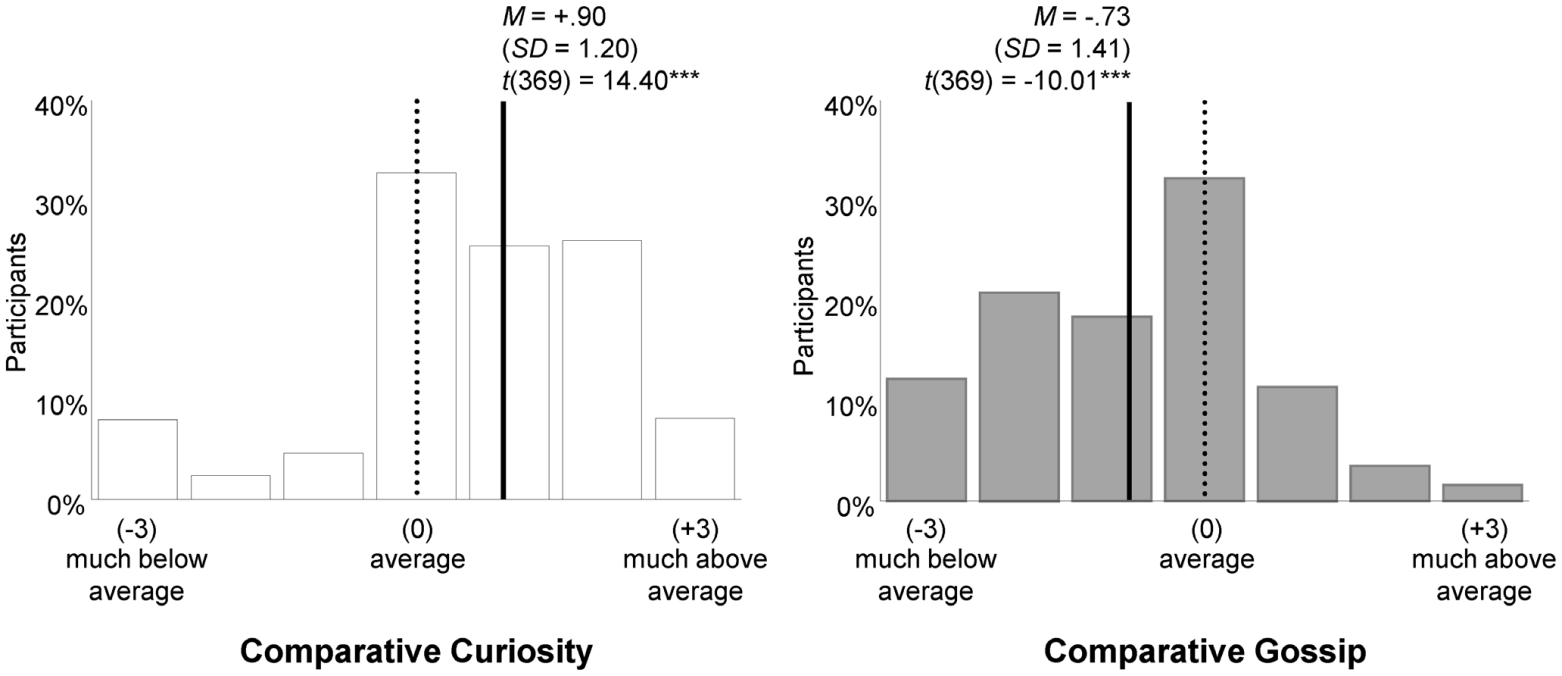
Self-ratings of Social Curiosity and Gossip (*N* = 370).

Participants rated themselves as being more curious, *M* = .90, *SD* = 1.20, *t* (369) = 14.40, *p*<.001, *d* = .75, but less gossipy than an average peer, *M* = −.73, *SD* = 1.41, *t* (369) = −10.01, *p*<.001, *d* = .52. As Figure 1 depicts, 60% of the participants rated their curiosity as being above average whereas more than 50% rated their tendency to gossip as below average. For both traits, being curious and being gossipy, no significant differences between the English and German sample emerged, *t* ´s (369) <1. Hence, participants appraised curiosity as a rather desirable trait whereas gossiping appeared to be viewed in a less positive way by the participants irrespective of whether they came from English speaking or German speaking countries.

In a next step, the relation between lay conceptions of curiosity and gossip and their relation with scientific trait conceptions of both concepts was examined. The nonsignificant correlations between self-rated curiosity and self-rated gossip, (comparative self-ratings: English sample: *r* = .10, German sample: *r* = .14, *p*’s >.09; absolute self-ratings: English sample: *r* = .09, German sample: *r* = .10, *p*’s >.17) suggest that the participants viewed the two concepts as two distinct personality attributes. To examine the discriminant validity of social curiosity and gossip lay conceptions, multiple regression analyses were computed with self-rated curiosity and self-rated gossip as dependent variable, respectively, as well as the trait measures for social curiosity (SCS-general, SCS-covert) and for gossip (GFQ-Information, GFQ-Friendship, GFQ-Entertainment, GFQ-Control) as independent variables. Since separate regression analyses of the two samples and for the two types of self-ratings (comparative and absolute) yielded highly similar results, only data for the total sample and the comparative self-ratings are reported. Self-rated comparative curiosity was only significantly related to the two trait social curiosity subscales, with adjusted *R^2^* = .18, *F*(6,363) = 14.15, *p*<.001. Participants who scored higher on the SCS-General (β = .32, *p*<.001) and the SCS-Covert subscale (β = .18, *p* = .001) viewed themselves as being more curious than their peers. The four GFQ subscales did not contribute significantly to regression (all β’s <.10, *ns.*). A similar picture emerged for self-rated comparative gossip: Participants’ view of their own tendency to gossip was significantly related to the four GFQ-subscales, with adjusted *R^2^* = .43, *F*(6,363) = 47.50, *p*<.001. The statistically most important predictor for self-rated comparative gossip was the GFQ-Entertainment subscale, β = .39, *p* = .001, followed by the GFQ-Friendship subscale, β = .18, *p*<.01, and the GFQ-Information subscale, β = .13, *p* = .02. The GFQ-Influence subscale was only marginally significant, β = .09, *p* = .07. Neither social curiosity subscales contributed to the regression, β’s <.06, *ns*.

### Structural Relationship between Measures of Social Curiosity and Gossip

**Figure 2:**
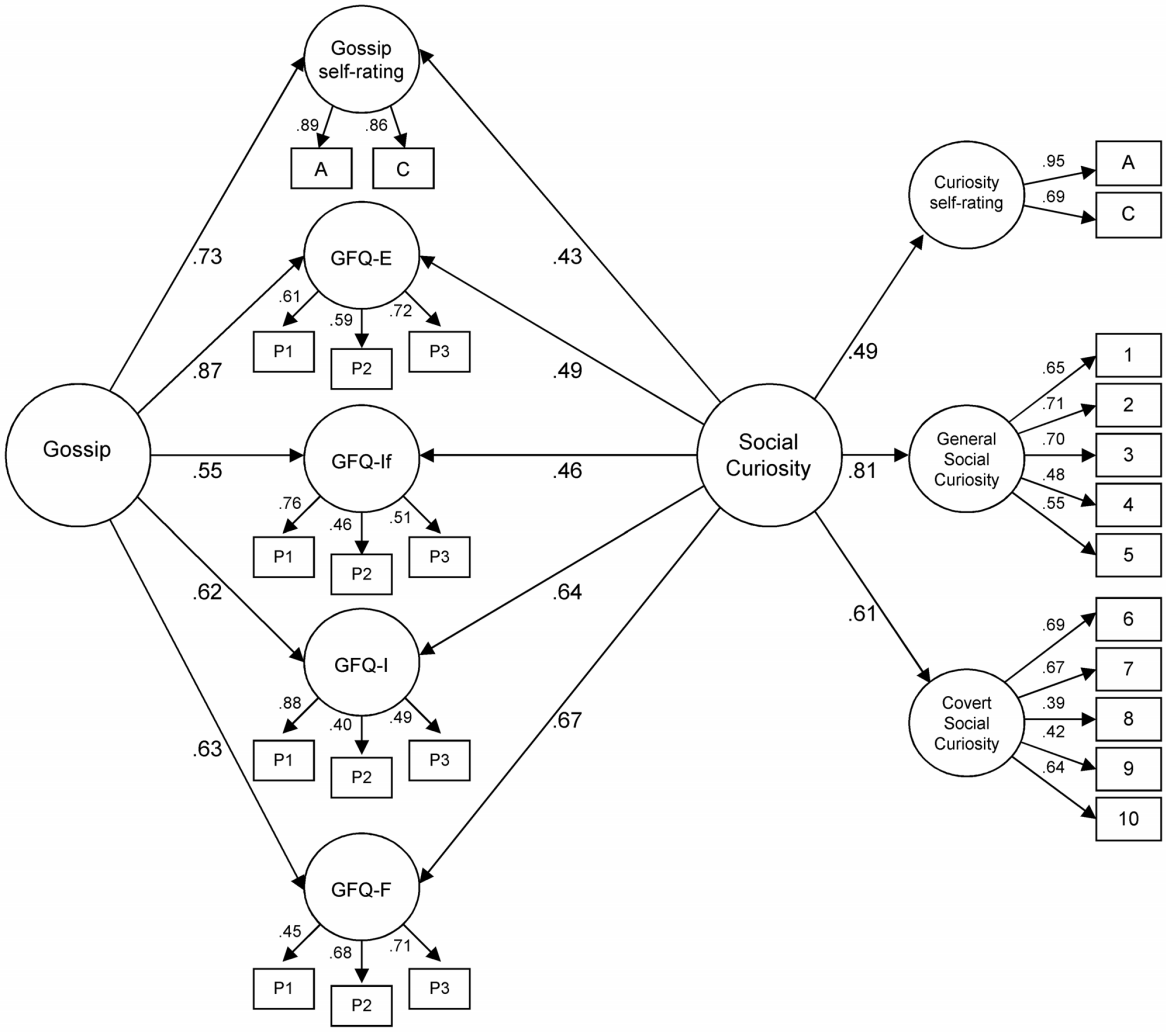
Standardized Factor Loadings and Inter-Factor Correlation for the Multidimensional Model (*N* = 370). SCS = Social Curiosity Scale; SCS-G = Subscale Social Curiosity-General; SCS-C = Subscale Social Curiosity-Covert; EC = Epistemic Curiosity Scale; CEI = Curiosity and Exploration Inventory – Trait Form; GFQ = Gossip Function Questionnaire; GFQ-I = Gossip Function Questionnaire-Information Subscale; GFQ-F = Gossip Function Questionnaire-Friendship Subscale; GFQ-If = Gossip Function Questionnaire-Influence Subscale; GFQ-E = Gossip Function Questionnaire-Entertainment Subscale; A = absolute self-rating; C = comparative self-rating.

In order to examine the structural relationship between social curiosity and gossip, confirmatory factor analyses (CFA) with maximum likelihood estimation were calculated using AMOS 17.0 [40]. Three different equivalent models were tested in order to examine the relationship between social curiosity and gossip. The first model represents the most parsimonious structural model consisting of a single-factor with paths to all 8 subscales (SCS-General, SCS-Covert, self-rated Curiosity, GFQ-Information, GFQ-Friendship, GFQ-Entertainment, GFQ-Influence, self-rated Gossip). Thus, Model 1 tested whether gossip represents one facet of a general social curiosity factor. The second model tested the hypothesized hierarchical CFA model with social curiosity and gossip as correlated second-order factors which were presumed to have direct effects on the respective subscales representing the three first-order factors for social curiosity (SCS-general, SCS-covert, self- rated Curiosity) and the five first-order factors for gossip (GFQ-Entertainment, GFQ-Friendship, GFQ-Information, GFQ-Influence, self-rated Gossip). Model 2 therefore tested the assumption whether social curiosity and gossip represent two distinct but related domains of interest. The third model replaces the correlation between the social curiosity and gossip factors with the specification that the five indicators for gossip are multidimensional (cf., Figure 2). Similar to the two-factorial model, the multidimensional model assumes that social curiosity and gossip are related but distinct phenomena. Extending the two-factorial model, however, it tested the structural relationship on the level of specific types of social functions (information gathering, facilitating social relationships, social control, and entertainment). In addition, all three structural models were compared to a null model which assumed that no factors were present in the data (cf. [41], [42]).

For all GFQ-scales, parcels were used to create indicators for latent variables within a structural equation approach. Parcels are sums or averages of two or more items of a construct. They have a lower error variance and are, thus, more reliable than single indicators [43]. For parcelling, random assignment method suggested by Little, Cunningham, Shahar, and Widaman [44] was used. The model fit was assessed by multiple goodness-of-fit (GOF) indices based on recommendations by Kline [41] and by Hu and Bentler [45]. The chi-squares and other GOF indices for each model are reported in Table 2. In a first step, all models were tested for the total sample, and, in a second step, invariance across groups was tested.

**Table 2:** Goodness-of-Fit Indicators for Models of Social Curiosity and Gossip.

Model	?^2^	*df*	?^2^ *diff*	*CFI*	*SRMR*	*RMSEA*	*90% CI*
*Total Sample (N = 370)*							
Null Model	3563.82	325	–	–	–	–	
1-Factor SC Model	832.40	293	2731.42	.83	.09	.071	.065–.076
2-Factor SC Model	777.13	290	55.27	.85	.08	.067	.062–.073
Multidimensional Model	757.75	286	19.38	.85	.08	.067	.061–.073
*English Sample (n = 218)*							
Null Model	2520.24	325					
1-Factor Model	745.44	293	1774.80	.79	.11	.084	.077–.092
2-Factor Model	688.97	290	65.47	.82	.08	.080	.072–.087
Multidimensional Model	666.10	286	22.87	.83	.09	.078	.071–.086
*German Sample (n = 152)*							
Null Model	1502.01	325	–	–	–	–	
1-Factor Model	503.92	293	998.09	.82	.10	.069	.059–.079
2-Factor Model	489.64	290	25.72	.84	.09	.068	.057–.078
Multidimensional Model	484.79	286	4.85	.83	.09	.068	.057–.078

*Note.* All *χ^2^* and all *χ^2^ diff* are significant at *p<*.001.

The chi-square statistics for the three models were significant (*p*<.01). The difference between the chi-squares for these models indicated that the multidimensional model had the smallest chi-square, χ^2^(286) = 757.75, *p*<.001, followed by the two-factor model with χ^2^(290) = 777.13, *p*<.001. The one-factor-model yielded the highest chi-square with χ^2^(293) = 832.40, *p*<.001. The CFI, SRMR, and RMSEA indices were within the acceptable range for the three models for the total sample as well as for the two subsamples (cf., Table 2). However, the GOF were better for the two-factorial model and the multidimensional model than for the one-factor model. As expected, the standardized factor loadings for both the two-factorial model and the multidimensional model were relatively high, ranging in magnitude from.43 to.97. All factor loadings were significant (*p*<.001). The two-factorial model yielded an inter-factor correlation high in size *(r* = .51), which suggests that gossip and social curiosity are related but different concepts. Moreover, the multidimensional model suggests that the interrelationships between social curiosity and the different facets of gossip varied in their strengths. The standardized factor loadings for the multidimensional model are presented in Figure 2. Social curiosity and gossip show both comparable high factor loadings on the subscales GFQ-Information and GFQ-Friendship (.62–.67). The factor loadings for the remaining gossip subscales (GFQ-Entertainment, GFQ-Influence, and self-rated Gossip), again show significant factor loadings for both gossip and social curiosity. However, all three gossip subscales yielded higher loadings on gossip (.55–.87) than on social curiosity (.43–.49).

In a next step, it was tested whether the factor loadings of gossip and social curiosity replicate across the two samples for the multidimensional model. Specifically, the initial two-group model in which no equality constraints were imposed was compared with a two-group model in which factorial loadings and measurement weights were constrained to be equal across both samples (cf. [42]). The GOF of the model for the two groups in combination and with no equality constraints imposed were satisfactory (CFI = .84; RMSEA = .05; SRMR = .08). The χ^2^ value, with 572 degrees of freedom, is 1150.92, p<.001. The model with the factor loading constrained to be equal across groups yielded a χ^2^(601) of 1184.56, p<.001. The two models did not differ significantly, χ^2^(33) = 33.64, ns, indicating that the factor loadings related to the multidimensional model were invariant. From the perspective of cross-validation, this illustrated equality serves as support for the multidimensional model.

In order to further explore the convergent and divergent validity of the found pattern, the relation of social curiosity (SCS) and gossip (GFQ) with trait curiosity (EC, CEI) and personality measures (N, E, O) was examined. Comparing the German and the English sample with respect to these measures yielded only three significant differences (cf., Table 1): The German sample scored significantly higher than the English sample on EC and Openness but lower on Neuroticism, all t’s >3, p<.001, d’s >.35. Since separate analyses of the two samples yielded highly similar results, only data for the total sample are reported.

Social curiosity as measured by the SCS correlated significantly with both trait curiosity scales (EC, CEI) and with the Openness scale providing evidence for convergent validity (cf., Table 3). Thus, participants high in social curiosity also scored higher on epistemic curiosity and appetitive strivings for novelty and challenge as well as demonstrating more openness to experience. Conversely, gossip as measured by the GFQ showed no significant correlation with these trait curiosity measures or openness, indicating divergent validity of social curiosity and gossip.

**Table 3:** Correlations between Curiosity, Gossip, and Personality Measures for the Total Sample (*N* = 370).

	Social Curiosity Scale (SCS)	Gossip Function Questionnaire (GFQ)
Trait Curiosity		
Epistemic Curiosity (EC)	.28***	.07
Curiosity and Exploration Inventory (CEI)	.30***	.04
NEO		
Neuroticism	.09	.20***
Extraversion	.28***	.25***
Openness	.24***	.01

*Notes*: ***p<.001.

Turning to Extraversion yielded significant positive correlations with the SCS, consistent with the notion that social curiosity and extraversion overlap to some extent. A similar pattern of results emerged for the GFQ, suggesting that the various means of gossiping are associated with higher levels of extraversion and negative affectivity.

## Discussion

The main goal of the present study was to examine the relationship between social curiosity and gossip. Lay conceptions of social curiosity and gossip indicate that both constructs represent differently evaluated and independent aspects of social behavior. Examining the relationship between trait conceptions of social curiosity and gossip also indicates that they represent distinct yet related domains of interest. In particular, social curiosity and gossip overlap in terms of social functions such as learning and relationship building.

### Lay-Conceptions of Social Curiosity and Gossip

This study is the first to investigate lay conceptions and trait conceptions of social curiosity and gossip. Participants from both the German and English samples uniformly perceived themselves as being more curious but less gossipy than their average peer. Thus, participants showed biased perceptions in both cases, since the average cannot be above or below average by definition. This pattern might indicate a social desirability bias for positive qualities like curiosity and negative qualities like gossiping [21], [46].

Lay conceptions of social curiosity and gossip were significantly related to respective trait conceptions as used in previous research. Self-rated social curiosity was significantly related to both facets of trait social curiosity (cf. [15]). Conversely, self-rated gossip showed the strongest relationship with the trait subscale “GFQ-Entertainment”, indicating that participants predominantly conceptualize conversations as gossip when they serve the purpose of pleasure and amusement (cf. also [47]). Social exchange in order to foster social relationships or gather information appears to represent also a substantial, however, less pronounced facet of lay conceptions of gossip. Social exchange as an informal social mechanism for controlling free riders and social cheats, the fourth facet of trait conceptions of gossip, was not significantly related to lay conceptions of gossip. This pattern indicates that the everyday understanding of the term gossip is narrower than and different from the construct used by researchers [2]. Accordingly, the discrepancy between observational and self-report studies on the frequency of gossip may partly be due to differences in the understanding of the term gossip.

### Trait and Self-Conceptions of Social Curiosity and Gossip

Analyzing trait and lay conceptions of gossip and social curiosity conjointly showed a more complex picture. Specifically, gossip and social curiosity appear to be two substantially related concepts, indicated by an intercorrelation of.51 between both factors. Similarly, Litman and Pezzo showed that interpersonal curiosity was moderately related to the tendency to gossip [48]. Thus, the interest in social conversations is a strong interlink between both aspects of a social behavior. Moreover, both constructs were substantially and similarly related to extraversion, indicating that curiosity and gossip are both rooted in sociability to some extent. However, the multidimensional model suggests that curiosity and gossip steer social conversation on the basis of different motive patterns. Gossip behavior appears to be more strongly driven by the desire for entertainment whereas social curiosity appears to be more strongly driven by a general interest in gathering information about how other people feel, think, and behave and the need to belong. This suggests that gossip represents more than an exploratory behavior in the harness of social curiosity and that social curiosity is more than a motivational ingredient of gossip. Divergent validity was also demonstrated in relation to other curiosity and personality measures. Only social curiosity was related to measures assessing curiosity in the realms of general knowledge and information acquisition (EC, CEI) and to openness to experience. Our findings are in line with previous research showing that epistemic curiosity overlaps curiosity for social information but only marginally overlaps gossip [48]. Thus, social curiosity and gossip represent two related but distinct aspects of social behavior suggesting that social conversations about other people may serve various needs. The pattern of results was equivalent across the two samples, providing additional support for divergent validity of social curiosity and gossip.

### Social Curiosity and Gossip: Related but Different Aspects of the Cultural Animal

Recent theoretical conceptions of human functioning such as the “cultural animal” conception by Baumeister [3], [49], stress the idea that humans are designed by nature to participate in and belong to a community and culture. Specifically, Baumeister argues that humans are adapted to live in a cultural society which enables individuals to store and share knowledge collectively, divide labour, and rely on others, rather than their own experience, for learning. In order to function in a constantly changing social world, cultural animals need to learn the culture’s knowledge and the rules for behavior in the society. Thus, negotiating our way through a complicated social and cultural environment is one of the major human tasks which require high plasticity for learning and adaptation throughout the lifespan. Social curiosity and gossip may represent major tools for promoting such a lifelong learning process. First results support the notion that the willingness to learn remains high across the lifespan. Results from a longitudinal study showed that social and epistemic curiosity was not affected by aging [50]. Similarly, Renner [15] found comparably high levels of social curiosity in both younger and older adults although younger adults reported a somewhat higher level of social curiosity. Presumably, the tendency to gossip also remains high across the lifespan, however, age graded results are still awaiting research.

Considering the present results, one may further speculate that social curiosity and gossip represent two different core drives of cultural learning. Information about other people and their behavior gives us the possibility to learn where pitfalls and opportunities lie without the need to learn from our own trials and errors (cf., social learning theory; [3], [51]). Social curiosity represents the basic motivational-behavior system which drives the general interest in the social world. The gathering and processing of information on other people enables individuals to effectively adapt to their social environment. However, in order to form a cultural system which stores and transmits knowledge between individuals and generations, they need to disseminate this information within their social environment. The need to gather social information and the need to disseminate social information might represent the two sides of the cultural learning coin (cf. [3], [4], [52]). Thus, the interest in those around us and the pleasure we derive from gossiping and transmitting information might ensure a continuing learning and adaptation process across the lifespan. In line with this notion, recent research from lifespan psychology suggests that high social activity promotes better cognitive functioning in older age [53]. This underscores that social participation is a fundamental prerequisite for human functioning (e.g. [2], [4], [23], [54]). Hence, people may be designed as cultural animals as suggested by Baumeister [3] with social curiosity and gossiping representing innate drives facilitating socialization and cultural fitness.
